# The fibroblast-driven melanoma/Treg vitiligo mouse model is effectively suppressed by IFNγ blocking antibody

**DOI:** 10.3389/fmed.2025.1677626

**Published:** 2025-09-26

**Authors:** Yuhua Xie, Jun Cui, Yanhua Lu, Yingxue Du, Jianmin Chang

**Affiliations:** ^1^Department of Dermatology, Beijing Hospital, National Center of Gerontology, Institute of Geriatric Medicine, Chinese Academy of Medical Sciences, Beijing, China; ^2^National Institute of Biological Sciences, Beijing, China

**Keywords:** melanoma/Treg vitiligo mouse model, bispecific antibody, fibroblast, IFNγ, vitiligo

## Abstract

The use of immunosuppressive drugs for vitiligo treatment carries a potential risk of increased infection, whereas IFNγ bispecific antibodies represent an alternative therapeutic strategy. Researchers employing the melanoma/Treg vitiligo mouse model identified a critical role of dermal fibroblasts during the progressive phase of vitiligo. These fibroblasts respond to IFNγ and recruit CD8 + T cells, a mechanism that also contributes to the bilateral symmetrical depigmentation commonly observed in vitiligo patients. To evaluate the suitability of this model as a platform for developing fibroblast-targeted IFNγ bispecific antibodies, mice were administered regular injections of IFNγ monoclonal antibodies. The IFNγ monoclonal antibody treatment significantly suppressed vitiligo progression in the melanoma/Treg model. Owing to its advantages—including straightforward induction, multi-antigen specificity, and the demonstrated efficacy of IFNγ blocking antibodies, the melanoma/Treg vitiligo mouse model is a robust platform for advancing the development of fibroblast-targeted IFNγ bispecific antibodies.

## Background

Immunosuppressive agents are increasingly pivotal in vitiligo management, functioning by attenuating immune-mediated melanocyte destruction (1, 2). However, this strategy compromises host defense, increasing the risk of infections and impairing antitumor immunity (3, 4). Bispecific antibodies (BsAbs), capable of simultaneously binding two antigens, offer the potential to remodel the local immune microenvironment and thus represent a promising approach in immunotherapy (5, 6). Bispecific antibodies therefore hold significant therapeutic potential in the treatment of vitiligo (7–14).

Our preliminary studies identified dermal fibroblasts in vitiligo-affected skin as critical orchestrators of CD8 + cytotoxic T-cell recruitment and activation through IFNγ-dependent chemokine secretion (15). Murine models reveal that region-specific IFNγ-resistant fibroblasts dictate autoimmune depigmentation patterns. Anatomically distinct fibroblast subpopulations exhibit divergent capacities to modulate IFNγ responses, a key determinant of vitiligo lesion distribution. These findings underscore fibroblasts as promising therapeutic targets, with IFNγ-targeting BsAbs emerging as a viable strategy.

We employed a novel melanoma/Treg vitiligo murine model that recapitulates hallmark clinical features: epidermal depigmentation, CD8 + T-cell infiltration, and melanocyte loss (15, 16). This model activates endogenous melanocyte-reactive CD8 + T cells through transient inoculation of B16F10 melanoma cells and depletion of CD4 + regulatory T cells. Compatible with all C57BL/6 strains using commercially available reagents, it provides a robust platform for mechanistic studies and drug discovery.

To address therapeutic limitations, fibroblast-targeted IFNγ BsAbs represent a promising alternative. To validate the melanoma/Treg model’s utility for BsAb development, we administered periodic IFNγ monoclonal antibody (mAb) injections. These mAbs significantly suppressed vitiligo progression, demonstrating sustained efficacy. Given the model’s technical simplicity, multi-antigen specificity, and responsiveness to IFNγ blockade, it serves as an optimal platform for advancing fibroblast-targeted IFNγ BsAb therapeutics.

## Questions addressed

In this study, we investigated whether the progression of vitiligo in the melanoma/Treg-induced vitiligo model could be inhibited by IFNγ blocking antibodies.

## Experimental design

### Mice

C57BL/6 N mice were purchased from Charles River Laboratories. Pmel TCR transgenic mice (stock no. 005023) was obtained from The Jackson Laboratory (17). Mice were bred and maintained in a specific pathogen-free facility in accordance with the Guide for the Care and Use of Laboratory Animals of the National Institutes of Biological Sciences (NIBS).

### Melanoma/Treg-induced vitiligo model

The melanoma/Treg-induced vitiligo model was established as previously described (16). Briefly, 8-9-week-old C57BL/6 mice were injected intradermally with B16F10 melanoma cells into the right flank (day 0). CD4^+^ T cells were depleted by intraperitoneal administration of anti-CD4 antibody on days 4 and 10. The primary tumor was surgically removed on day 12 to prevent tumor overgrowth and to promote the activation of melanocyte-reactive CD8^+^ T cells. Mice were subsequently monitored for the development of depigmentation and immune cell infiltration in the skin.

### Cell isolation and culture

For isolation of mouse primary dermal fibroblasts, the dorsal skin of C57BL/6 N newborns (P1–P3) were flattened and placed dermis-side down in 2.4 U/mL dispase (Gibco) solution overnight at 4 °C, then epidermis were removed from dermis and the dermis were collected and placed in 2 mg/mL collagenase (Sigma-Aldric) at 37 °C with shaking at 80 rpm for 1 h, centrifugated and resuspended the dermal pallet and filtered with 40 μm filter, the single-cell suspension was maintained in DMEM (Gibco) supplemented with 10% (v/v) FBS (HyClone), 1% (v/v) pen–strep (Gibco), 1 × antibiotic–antimycotic (Gibco), and were cultured at 37 °C in a cell incubator with 5% CO2.

### RNA extraction and qPCR

The medium was removed from cultured primary fibroblasts and added with Trizol (Thermo Fisher Scientific) and RNA extraction was conducted using the Direct-Zol RNA Miniprep Kit (Zymo Research), according to the manufacturer’s instructions. Then equal amounts of RNA were added to reverse-transcriptase reaction mix (Vazyme) to obtain cDNA. Expression levels were normalized to the expression of PPIB (15, 18). qPCR was conducted using a CFX96TM Real-Time system (Bio-Rad) with SYBR FAST qPCR Kit (KAPA). All primer pairs were designed for the same cycling conditions: 10 min at 95 °C for initial denaturation, 40 cycles of 10 s at 95 °C for denaturation, 30 s at 62 °C for annealing, and 10 s at 65 °C for extension. The primers were designed to produce a product spanning an exon-intron boundary in each of the target genes. CXCL9 forward primer sequence (5′-3′): AACGGAGATCAAACCTGCC, reverse primer sequence (5′-3′): GGGTGTTTTGGGTTTTCTGTT. CXCL10 forward primer sequence (5′-3′): TCAGCACCATGAACCCAAG, reverse primer sequence (5′-3′): CTATGGCCCTCATTCTCACTG. PPIB forward primer sequence (5′-3′): AGCAAGTTCCATCGTGTCATC, reverse primer sequence (5′-3′): AGCTTGAAGTTCTCATCTGGG. For qPCR data analysis, relative gene expression was calculated using the comparative Ct (ΔΔCt) method. For each sample, the Ct value of the target gene was normalized to the internal reference gene PPIB to obtain ΔCt. ΔΔCt values were derived by comparing the ΔCt of experimental samples with that of the designated control group. Relative expression levels were expressed as fold change and calculated as 2^−ΔΔCt.

### Antibody biodistribution characterization

**Figure 1:**
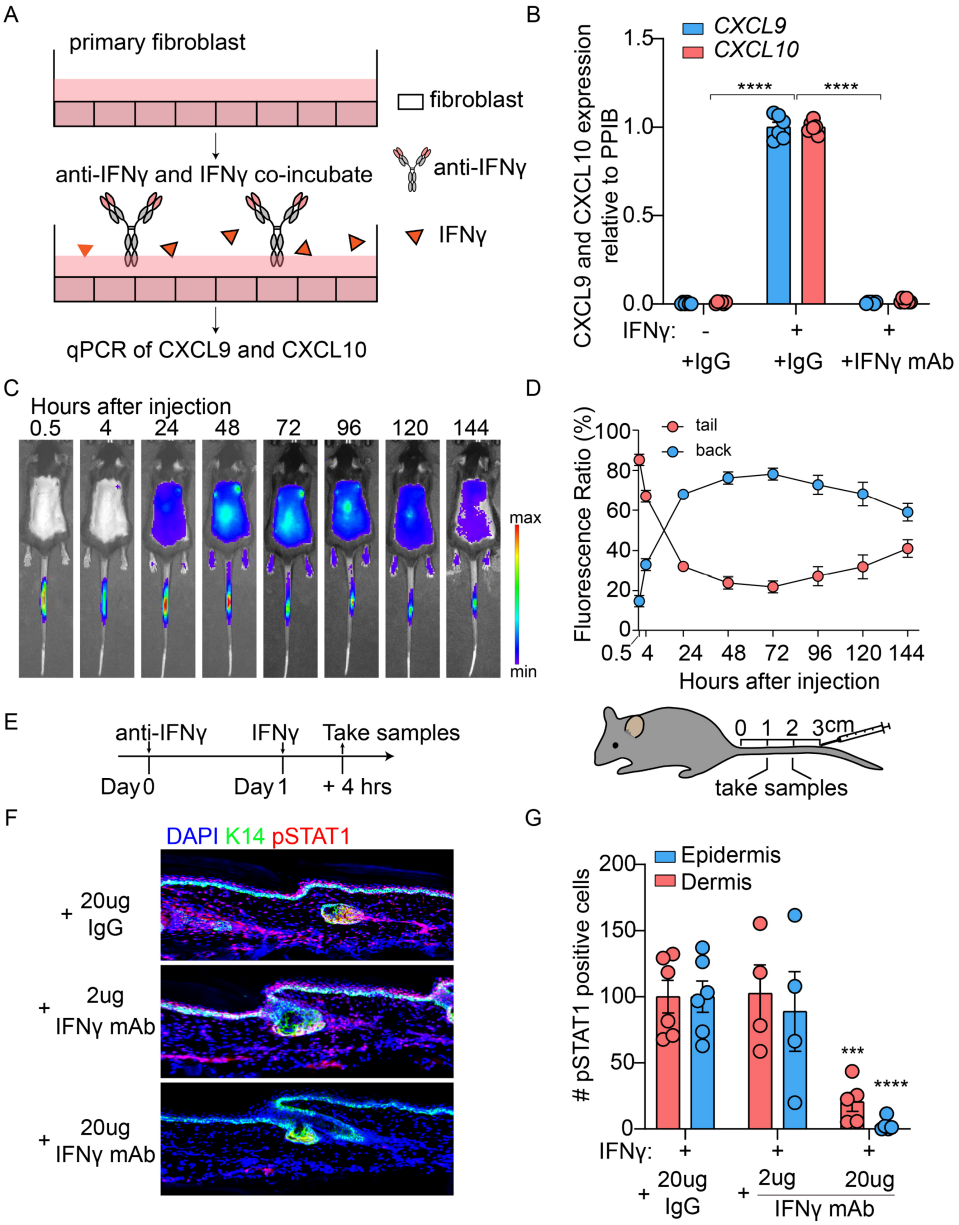
IFNy blocking antibody directly inhibited the downstream signaling pathways activated by IFNy both *in vitro* and *in vivo*. **(A)** Schematic diagram of in vitro treatment for primary fibroblasts with IFNy monoclonal antibody (mAb). **(B)** CXCL9/10 mRNA levels in primary fibroblasts stimulated with IFNy ± IFNy mAb, quantified by qPCR (*n*=6). **(C,D)** Whole body IVIS fluorescence images **(C)** and graph calculating radiant efficiency of IVIS imaged back/tail of mice are shown (**D**, *n*=4). **(E)** Schematic diagram of local IFNy challenge + IFNy mAb pre-treatment in tail skin. **(F)** Representative immunofluorescence images of pSTAT1+ cells in epidermal and dermal layers. **(G)** Quantification of pSTAT1+ cells numbers in epidermis and dermis (*n* = 6).

The antibodies were labeled with Cy-7 using Cy^®^7 Mono NHS Ester kit (GE), performed according to the manufacturer’s instructions. Cy-7 labeled antibodies were administered by an intradermal injection of 20 μg into mice tails with a single dose. Mice under isoflurane anaesthesia were fluorescently imaged using the following excitation and emission filters: 745/800 nm for Cy-7. Epi-fluorescent images were acquired using an IVIS Lumina III Imaging System (PerkinElmer) at 0.5/4/24/48/72/96/120/144 h post antibodies administration. Regions of interest (ROIs) were drawn in the defined area and quantified in the physical, calibrated unit “Radiant Efficiency [p/s/sr]/[μW/cm^2^]”. For the fluorescence ratio analysis, our experimental aim was to determine the retention time of IFNγ mAb in mouse tails, in order to define the experimental time window. Therefore, we quantified fluorescence separately in the back and the tail. The decrease in tail fluorescence together with the increase in back fluorescence reflected the diffusion and residual amount of IFNγ mAb in the tail skin. The y-axis of Figure 1D (%), represents: tail fluorescence/(tail fluorescence + back fluorescence) × 100.

### IFNγ challenge

For *in vitro* experiment, mouse primary fibroblasts were plated in 24-well culture plates at 150000 cells per well, 24 h later, the medium was changed and cells were incubated with 40 U/mL mouse IFNγ (GenScript) and 2 μg/mL IFNγ monoclonal antibodies simultaneously for 3 h. The IFNγ monoclonal antibody used was InVivoMAb anti-mouse IFNγ (cat. BE0055, Rat IgG1, BioXcell). The negative control group (−) received the corresponding isotype control IgG, Rat IgG1 (*κ*), BioXcell, as recommended by the manufacturer. Cells were collected for CXCL9 and CXCL10 qPCR analysis. For the *in vitro* experiments, six independent biological replicates were conducted. In each experiment, two technical replicates (wells) were set up for each experimental condition. Each independent experiment yielded one result, and the aggregated data from the six experiments were used to generate the statistical plot in Figure 1B.

For *in vivo* experiment, the IFNγ monoclonal antibody used was InVivoMAb anti-mouse IFNγ (cat. BE0055, Rat IgG1, BioXcell). The negative control group (−) received the corresponding isotype control IgG, Rat IgG1 (*κ*), BioXcell. Twenty-four hours prior to the induction, the corresponding antibodies were administered via intradermal injection into the tail of wild-type mice. The control group (−) received 25 μg Rat IgG1 (κ), while the experimental groups received 2 μg and 25 μg IFNγ monoclonal antibody, respectively. Then one day later, 20 ng mouse IFNγ was administered by an intradermal injection into mice tails at the same site. And 4 h later, the tail skins were collected for section staining. In the 2 μg IFNγ mAb group, one mouse died due to excessive gas anesthesia, and therefore samples were collected from four mice. For the other two experimental conditions, samples were collected from six mice each.

### IFNγ mAb treatment in vitiligo mouse model

In Pmel mice, vitiligo develops progressively between postnatal day 20 and day 50, and therefore we administered IFNγ mAb every other day during this period. In the melanoma/Treg-induced vitiligo model, IFNγ mAb administration was initiated on day 12 after vitiligo induction and continued every other day until day 33. The antibody used was the InVivoMAb anti-mouse IFNγ monoclonal antibody (BioXcell). For the control group (anti-IFNγ [−]), we employed the manufacturer-recommended isotype control IgG, Rat IgG1 (*κ*). Antibodies were prepared in PBS at a concentration of 1 mg/mL. Specifically, the anti-IFNγ (−) group received 250 μg isotype control IgG in 250 μL; the anti-IFNγ (++) group received 250 μg IFNγ mAb in 250 μL; and for the anti-IFNγ (+) group, IFNγ mAb was diluted in PBS to a concentration of 80 μg/mL, with 20 μg administered in 250 μL. Each experimental condition was applied to *n* = 6 mice.

To account for potential effects of non-specific antibody administration, the control groups consistently received isotype control IgG. Additionally, IFNγ mAb injections diffuse extensively along the tail; therefore, sampling was performed at regions distant from the injection sites to avoid local effects. To prevent repeated injections at the same site, previous injection points were marked, and subsequent injections were administered at different locations. Through these optimizations, we observed no skin damage or other abnormalities at the sampling sites.

### Immunofluorescent staining

The mice tail skin tissues were embedded in O.C.T. compound, frozen and cryo-sectioned (25 μm). Sections were fixed for 10 min in 4% (v/v) paraformaldehyde (Thermo Fisher Scientific) at room temperature, permeabilized for 20 min in 3% H2O2 in methanol at −20 °C, blocked for 1 h in blocking buffer (2% normal donkey serum, 1% BSA, and 0.5% Triton in PBS). The anti-phospho-Stat1 were incubated overnight at 4 °C, then performed by the procedure of Tyramide signal amplification (TSA) technology. After that, anti-K14 were incubated 1 h at room temperature, then the secondary antibodies were incubated at room temperature for 1 h. For whole mount staining, the tail skin was carefully excised and cut into small pieces measuring approximately 1 cm × 0.5 cm. The tissue samples were then incubated in 25 mM EDTA at 37 °C for 2 h on a shaker set to 150 rpm. Following this treatment, the epidermis, along with attached hair follicles, was gently peeled away from the dermis using fine forceps. The isolated epidermal sheets were fixed with 4% paraformaldehyde for 7 min, rinsed thoroughly with PBS three times, and, when needed, extended anagen-stage hair follicles that obstructed visualization of shorter follicles were carefully removed under a stereomicroscope. Finally, the samples were subjected to immunofluorescence staining, followed by microscopic imaging. Immunofluorescence staining slides were imaged on a Nikon AX confocal microscope. Microscopy data were analyzed using Bitplane Imaris. Anti-phospho-Stat1 (CST, Cat.9167, Rb, 1,000x), anti-K14 [lab made (19), Rt, 1,000x], anti-DCT [lab made (15), Rb, 2000x] and anti-CD8 (ebioscience, Cat:17-0081-83, Rt, 500x) were used. Donkey anti-Rabbit IgG (H+L) Alexa Fluor 488 (Jackson ImmunoResearch, Cat.711-545-152, 1,000x), Donkey anti-Rabbit IgG (H+L) Alexa Fluor 555 (Jackson ImmunoResearch, Cat.711-565-152, 1,000x), Donkey anti-Rat IgG (H+L) Alexa Fluor 488 (Jackson ImmunoResearch, Cat.712-546-150, 1,000x), Donkey anti-Rat IgG (H+L) Alexa Fluor 555 (Jackson ImmunoResearch, Cat.712-565-150, 1,000x) were used.

### Statistical analysis

Statistical and graphical analyses were performed with GraphPad Prism, version 9.0.0. All data are shown as mean ± SEM. Ordinary one-way ANOVA or unpaired Student’s t tests were used for comparisons between groups. *p < 0.05, **p < 0.01, ***p < 0.001, ****p < 0.0001; NS, *p*>0.05, not significant.

## Results

### IFNγ blocking antibody directly inhibited the downstream signaling pathways activated by IFNγ both *in vitro* and *in vivo*

To investigate whether IFNγ antibodies can inhibit the melanoma/Treg-induced vitiligo model, we first explored the treatment conditions for directly inhibiting IFNγ using an IFNγ mAb both in vitro and in vivo. Primary fibroblasts were cultured in the presence of IFNγ with or without the IFNγ mAb (Figure 1A). After 24 h, qPCR was performed to assess CXCL9 and CXCL10 mRNA levels (Figure 1B). Compared to the control group, primary fibroblasts treated with IFNγ alone showed significantly elevated CXCL9 and CXCL10 mRNA levels. Co-treatment with 2 μg of the IFNγ monoclonal antibody markedly reduced these mRNA levels, bringing them close to those observed in the control group.

To evaluate the efficacy of the IFNγ mAb *in vivo*, we conducted intradermal injections of IFNγ and the IFNγ monoclonal antibody into the tail skin of wild-type mice. Monitoring at different time points revealed that the IFNγ mAb signal remained high for up to 4 h post-injection and retained approximately one-third of its initial intensity after 48 h (Figures 1C,D). In addition, the signal of the IFNγ monoclonal antibody gradually became detectable in the dorsal skin, indicating its diffusion to this site. Following intradermal pre-treatment with IFNγ mAb in tail skin, IFNγ challenge was administered 24 h post-injection. Tissue samples were harvested after a 4-h incubation period for pSTAT1 signaling assessment (Figure 1E). Immunohistochemical analysis revealed robust pSTAT1 activation across both epidermal and dermal compartments in IFNγ-only treated specimens. In contrast, IFNγ mAb pre-treatment induced marked attenuation of pSTAT1 signaling intensity in these layers (Figures 1F,G).

### The IFNγ blocking antibody significantly suppressed the progression of vitiligo in the Pmel model in a dose-dependent manner

**Figure 2:**
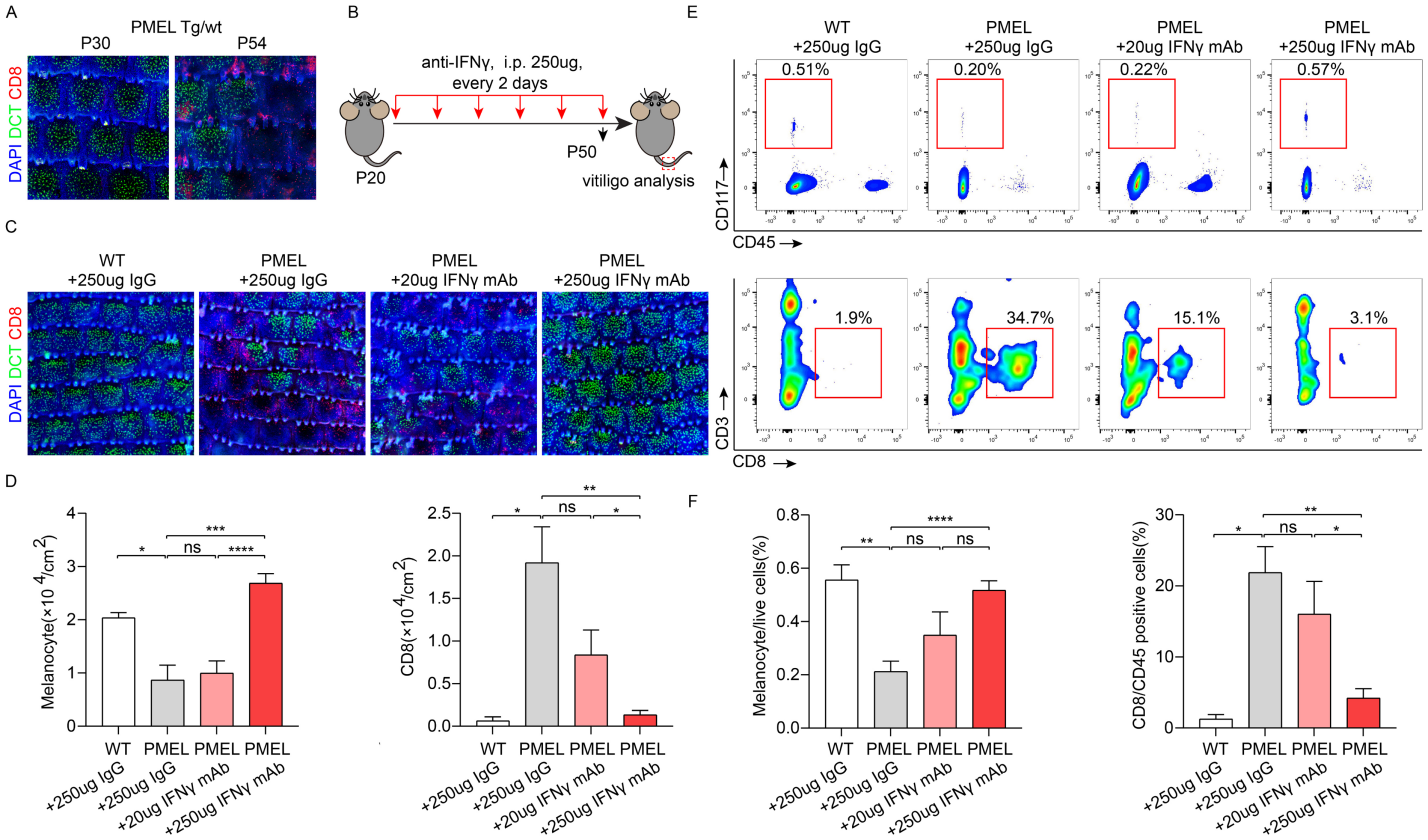
The IFNy blocking antibody significantly suppressed the progression of vitiligo in the Pmel model in a dose-dependent manner. **(A)** Representative wholemount immunofluorescence images of Pmel mouse skin at indicated time points. DCT marks melanocytes: CD8+ labels cytotoxic T cells. **(B)** Schematic diagram of IFNy monoclonal antibody (mAb) administration in Pmel mice. **(C)** Wholemount staining of Pmel skin following IFNy mAb treatment. **(D)** Quantification of melanocytes (DCT+) and CD8+ T cells in treated skin (*n* = 6). **(E)** Representative images of flow cytometry analysis. Melanocytes (CD117+ CD45−) and CD8+ T cells (CD3+ CD8+). **(F)** Statistical analysis of flow cytometry data (*n*=6).

Before applying our findings to the melanoma/Treg model, we optimized the dosing regimen of the IFNγ mAb using the established Pmel model (17, 20). In this model, Pmel mice develop vitiligo phenotypes between postnatal days 20 and 50 (Figure 2A). Therefore, from day 20 to day 50, mice received intraperitoneal injections of the IFNγ mAb every two days (Figure 2B). On day 50, whole-skin tissue staining and flow cytometric analysis revealed that the IFNγ mAb effectively inhibited vitiligo progression in the Pmel model (Figures 2C–F). There were more melanocytes in the epidermis, fewer infiltrating CD8+ T cells, and a dose-dependent effect, with 250 μg of the IFNγ mAb showing the most significant inhibition of disease progression.

### The progression of vitiligo in the melanoma/Treg-induced model was significantly suppressed by IFNγ blocking antibody

**Figure 3:**
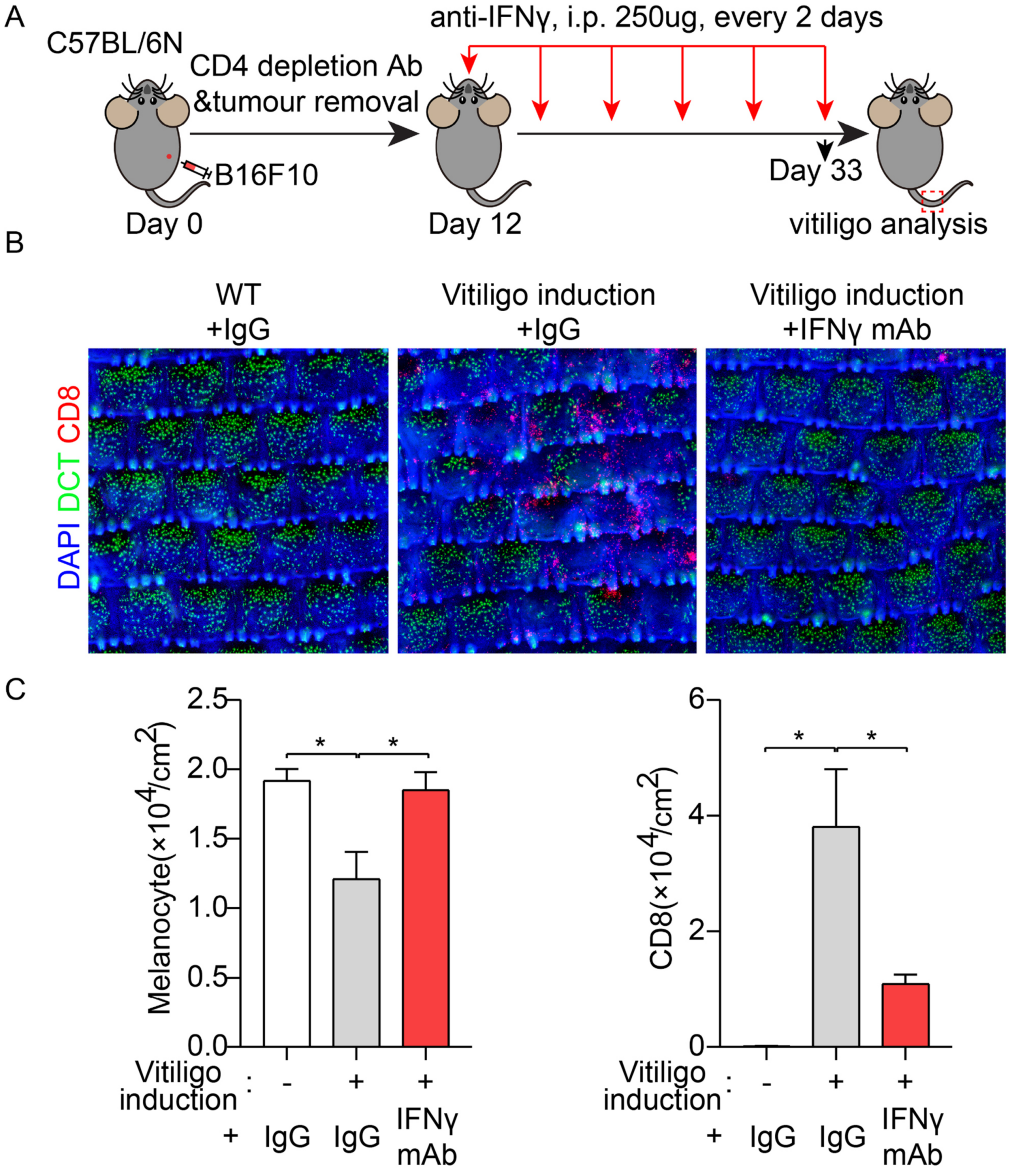
The progression of vitiligo in the melanoma/Treg-induced model was significantly suppressed by IFNy blocking antibody. **(A)** Schematic diagram of induction of melanoma/Treg vitiligo model and IFN y monoclonal antibody (mAb) administration. **(B)** Wholemount immunofluorescence imaging of melanoma/Treg vitiligo mice & IFNy mAb. DCT marks melanocytes; CD8+ labels cytotoxic T cells. **(C)** Quantitative analysis of Wholemount immunofluorescence imaging of melanoma/Treg vitiligo mice ± IFNy mAb (*n*=6).

To determine whether the melanoma/Treg model could respond to IFNγ blocking antibodies to inhibit vitiligo progression, we induced the disease according to previously described conditions: Intradermal injection of melanoma cells into the right flank of 8-9-week-old C57 mice (day 0), followed by CD4 depletion on days 4 and 10, and surgical removal of the primary tumor on day 12 (Figure 3A). From day 12 to day 33, mice received intraperitoneal injections of 250 μg of the IFNγ mAb every two days. On day 33, tail skin was analyzed for vitiligo phenotype (Figures 3B,C). Whole-skin tissue staining revealed that normally induced mice exhibited a vitiligo phenotype characterized by a significant reduction in melanocytes and increased infiltration of CD8+ T cells. In contrast, treatment with the IFNγ mAb significantly attenuated the vitiligo phenotype in the melanoma/Treg model.

## Conclusions and perspectives

Immunosuppressive agents have assumed growing therapeutic significance in vitiligo management, attenuating melanocyte-targeted autoimmunity through systemic immune suppression. However, this pharmacological strategy inherently compromises pathogen defense mechanisms, elevating infection susceptibility. BsAbs enable localized remodeling of the immune microenvironment and represent a highly promising therapeutic strategy for vitiligo.

Our preliminary investigations revealed that dermal fibroblasts critically orchestrate CD8+ T-cell recruitment and activation in vitiligo pathogenesis through chemokine secretion (15). Region-specific IFNγ-resistant fibroblasts were identified as determinants of autoimmune depigmentation patterning, with anatomically distinct subpopulations exhibiting differential capacities to modulate IFNγ responses. Building upon these findings, fibroblast-targeted IFNγ BsAbs emerge as a promising therapeutic alternative for localized immunomodulation.

On the other hand, although systemic neutralization of IFNγ may theoretically alleviate inflammatory responses in autoimmune diseases such as vitiligo, it also carries considerable drawbacks and risks (21). IFNγ is essential for antiviral, antibacterial, and antifungal immunity, and its systemic neutralization increases susceptibility to infections (21). Moreover, IFNγ enhances antigen presentation and activates NK cells and CD8^+^ T cells, thereby contributing to antitumor immunity; systemic blockade of IFNγ can therefore impair antitumor immunity (22). As an alternative, local administration of IFNγ mAb to neutralize IFNγ represents an alternative approach; however, this approach may still be limited by antibody diffusion. Indeed, intradermal injection of IFNγ mAb into the mouse tail resulted in systemic distribution (Figure 1C). Thus, local IFNγ neutralization remains associated with potential risks and limitations. In contrast, fibroblast-targeted IFNγ bispecific antibodies may offer a more promising therapeutic strategy for vitiligo.

In this study, we developed a melanoma/Treg murine model that faithfully recapitulates vitiligo hallmarks: epidermal depigmentation, CD8+ T-cell infiltration, and melanocyte loss. Periodic IFNγ mAb administration in this model significantly attenuated depigmentation progression, demonstrating sustained therapeutic efficacy. Notably, the model’s technical reproducibility, multi-antigen specificity, and responsiveness to IFNγ blockade establish it as an optimal preclinical platform for BsAb development. Subsequent investigations of fibroblast-targeted IFNγ BsAbs can leverage this system for efficacy screening and mechanistic exploration.

Compared with classical vitiligo models such as the Pmel mouse, the melanoma/Treg-induced vitiligo model offers several advantages. First, its hallmark features include a marked increase of CD8^+^ T cells and a reduction of Tregs within skin lesions, closely resembling the imbalance observed in vitiligo patients. Importantly, this model preserves multiple melanocyte-associated antigens, thereby better reflecting the antigenic diversity of human vitiligo. By contrast, CD8^+^ T cells in PMEL TCR transgenic mice express a single TCR specific for the melanocyte antigen gp100, thus lacking immunological diversity. Therefore, compared with the Pmel model, the melanoma/Treg-induced vitiligo mouse model more faithfully recapitulates the clinical condition of vitiligo patients. Consequently, it provides a more rational and broadly applicable platform for the development of next-generation therapeutic strategies. Second, previous studies employing this melanoma/Treg-induced model revealed that dermal fibroblasts act as the primary IFN-*γ*–responsive cells orchestrating CD8^+^ T-cell recruitment. This finding has also been validated in patient samples and explains the clinically common patterns of symmetrical and region-specific lesion distribution. Thus, for the development of fibroblast-targeted IFN-γ bispecific antibodies, the melanoma/Treg-induced vitiligo mouse model represents the most appropriate choice.

In summary, targeting IFN-γ with bispecific antibodies represents a highly promising therapeutic approach for vitiligo. Considering the principles of disease induction, the closer resemblance to patient pathology, and the central role of the IFN-γ pathway in fibroblasts, the melanoma/Treg-induced vitiligo mouse model is the most suitable preclinical platform for developing fibroblast-targeted IFN-γ bispecific antibodies.

## Data Availability

The raw data supporting the conclusions of this article will be made available by the authors, without undue reservation.
